# Endobronchial ultrasound-guided transbronchial needle aspiration combined with either endoscopic ultrasound-guided fine-needle aspiration or endoscopic ultrasound using the EBUS scope-guided fine-needle aspiration for diagnosing and staging mediastinal diseases: a systematic review and meta-analysis

**DOI:** 10.6061/clinics/2020/e1759

**Published:** 2020-10-05

**Authors:** Yanhua Shen, Shanyu Qin, Haixing Jiang

**Affiliations:** I Department of Endoscopy, The Affiliated Tumor Hospital of Guangxi Medical University, Nanning, Guangxi, China; II Department of Gastroenterology, First Affiliated Hospital of Guangxi Medical University, Nanning, Guangxi, China

**Keywords:** Endosonography, EUS-FNA, Mediastinal Diseases

## Abstract

The present systematic review and meta-analysis aimed to evaluate the available evidence base on endobronchial ultrasound-guided transbronchial needle aspiration (EBUS-TBNA) combined with either endoscopic ultrasound-guided fine-needle aspiration (EUS-FNA) or endoscopic ultrasound using the EBUS scope-guided fine-needle aspiration (EUS-B-FNA) for diagnosing and staging mediastinal diseases.

PubMed, Web of Science, and Embase were searched to identify suitable studies up to June 30, 2019. Two investigators independently reviewed articles and extracted relevant data. Data were pooled using random effect models to calculate diagnostic indices that included sensitivity and specificity. Summary receiver operating characteristic (SROC) curves were used to summarize the overall test performance.

Data pooled from up to 16 eligible studies (including 10 studies of 963 patients about EBUS-TBNA with EUS-FNA and six studies of 815 patients with EUS-B-FNA) indicated that combining EBUS-TBNA with EUS-FNA was associated with slightly better diagnostic accuracy than combining it with EUS-B-FNA, in terms of sensitivity (0.87, 95%CI 0.83 to 0.90 *vs.* 0.84, 95%CI 0.80 to 0.88), specificity (1.00, 95%CI 0.99 to 1.00 *vs.* 0.96, 95%CI 0.93 to 0.97), diagnostic odds ratio (413.39, 95%CI 179.99 to 949.48 *vs.* 256.38, 95%CI 45.48 to 1445.32), and area under the SROC curve (0.99, 95%CI 0.97 to 1.00 *vs.* 0.97, 95%CI 0.92 to 1.00).

The current evidence suggests that the combination of EBUS-TBNA with either EUS-FNA or EUS-B-FNA provides relatively high accuracy for diagnosing mediastinal diseases. The combination with EUS-FNA may be slightly better.

## INTRODUCTION

Mediastinal diseases can be caused by lung cancer, tuberculosis, sarcoidosis, inflammation, and other malignant tumors (1). Proper treatment and management of a mediastinal disease depends on accurate diagnosis and staging. Important minimally invasive methods for achieving this are endobronchial ultrasound-guided transbronchial needle aspiration (EBUS-TBNA) and endoscopy ultrasound-guided fine-needle aspiration (EUS-FNA), and combining the two is attractive because together they can cover nearly the entire mediastinum (2-5). More recently, EBUS scope-guided fine-needle aspiration (EUS-B-FNA) has emerged as a particularly convenient procedure (6,7). The combination of EBUS-TBNA and EUS-B-FNA can cover nearly the complete mediastinum and can be performed by one doctor using a single endoscope. International lung cancer staging guidelines recommend EBUS-TBNA combined with either EUS-FNA or EUS-B-FNA for diagnosing and staging mediastinal diseases (8-10).

To optimize such diagnosis and staging, we meta-analyzed the literature on the diagnostic accuracy of EBUS-TBNA combined with EUS-FNA or EUS-B-FNA.

## MATERIAL AND METHODS

To evaluate which method is better to combine with EBUS-TBNA and to provide a reference for clinical work, we searched PubMed, Web of Science, and Embase for studies that were published from January 2005 to July 2019 and that evaluated the accuracy of EBUS-TBNA combined with EUS-FNA or EUS-B-FNA for diagnosing and staging mediastinal diseases. Databases were searched using the following search string: (“endobronchial ultrasound-guided transbronchial needle aspiration” OR “EBUS-TBNA” OR “endobronchial ultrasonography” AND “endoscopy ultrasound-guided fine-needle aspiration” OR “EUS-FNA” OR “endoscopic ultrasound using the EBUS scope-guided fine-needle aspiration” OR “EUS-B-FNA” OR “endoscopic ultrasound using the EBUS bronchoscope” OR “transesophageal endoscopic ultrasound-guided needle aspiration” OR “transesophageal endoscopic ultrasound-guided fine-needle aspiration”) AND (“mediastinal disease” OR “mediastinal tumor”). Only original reports in English published in peer-reviewed journals were included (11), as long as they ① were a clinical trial or cohort study, irrespective of whether they were randomized or not, retrospective or prospective, ② compared EBUS-TBNA combined with EUS-FNA or EUS-B-FNA in patients with suspected mediastinal disease, irrespective of whether EBUS-TBNA was used first or second, and ③ reported sufficient data for calculating rates of true positives, false positives, true negatives, and false negatives.

We excluded studies if they ① were abstracts, reviews, comments, editorials, or studies involving fewer than 10 patients, ② sampled lesions outside the mediastinum, or ③ re-analyzed previously published data.

### Data extraction

Two authors independently reviewed abstracts initially, and then read the full text of potentially eligible studies. Reference lists in relevant articles were cross-checked to find additional potentially eligible articles. All articles ultimately included in the systematic review were read in full.

The same two authors independently extracted data from the included studies on the study population, diagnostic methods, and diagnostic outcomes, including sensitivity, specificity, and positive and negative predictive values.

### Statistical analysis

Data from each study were pooled and used to calculate the following indices of diagnostic accuracy: sensitivity, specificity, positive predictive value, negative predictive value, positive likelihood ratio, and negative likelihood ratio. Publication bias was assessed with funnel plots, which demonstrate the relationship between the sample size of the studies and the precision in estimating the outcome. Study heterogeneity was assessed using a random effect model, and *I^2^* was calculated to show the percentage of variability for between-study heterogeneity; *I^2^* >50% was deemed to represent substantial heterogeneity, and *p*<0.05 was defined as indicating significant heterogeneity (12-13). Analyses were carried out using meta disc1.4 and STATA 15.0.

## RESULTS

We included 16 studies involving 1,778 patients who were diagnosed with mediastinal diseases based on the combination of EBUS-TBNA with EUS-FNA (10 studies, 963 patients) or EUS-B-FNA (six studies, 815 patients) (Table 1). We failed to identify systematic reviews on these combination modalities. The Q value of heterogeneity test of the combination of EBUS-TBNA with EUS-FNA is 0.143, *I^2^*=0.0%, *p*>0.05, 95%CI (0.00 to 100.00); and the Q value of heterogeneity test of the combination of EBUS-TBNA with EUS-B-FNA is 30.948, *I^2^*=93.54%, *p*<0.01, 95%CI (87.58 to 99.19). It shows the apparent heterogeneity in the studies of the combination of EBUS-TBNA with EUS-B-FNA.

The pooling of data across the 10 studies using the combination of EBUS-TBNA with EUS-FNA indicated a pooled sensitivity of 0.87 (95%CI 0.83 to 0.90), with sensitivity in individual studies ranging from 0.68 to 1.00. The pooled specificity was 1.00 (95%CI 0.99 to 1.00), and specificity in the individual studies ranged from 0.98 to 1.00.

The pooling of data across the six studies using EBUS-TBNA with EUS-B-FNA indicated a pooled sensitivity of 0.84 (95%CI 0.80 to 0.88), with sensitivity in individual studies ranging from 0.68 to 0.96. The pooled specificity was 0.96 (95%CI 0.86 to 1.00), with specificity in individual studies ranging from 0.87 to 1.00.

**Figure 1:**
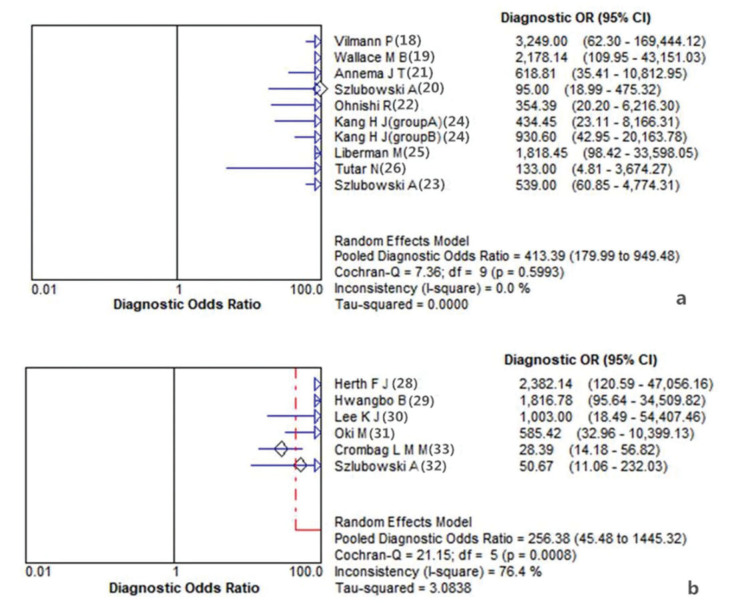
Forest plot of diagnostic odds ratio for included studies using the combination of EBUS-TBNA with EUS-FNA(a) or EUS-B-FNA(b).

**Figure 2:**
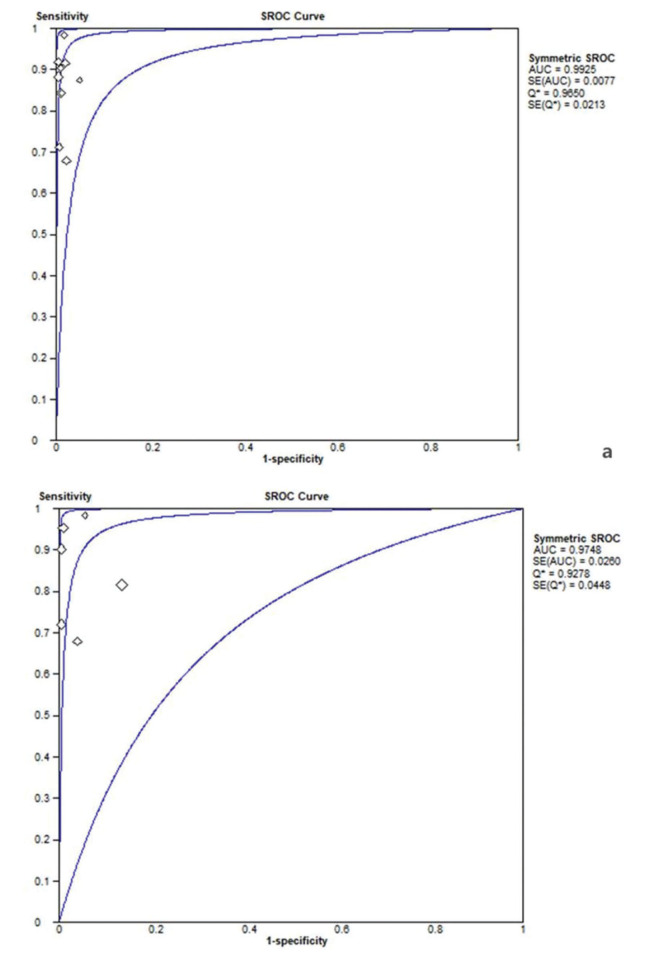
Summary receiver operating characteristic curve for data pooled from studies using the combination of EBUS-TBNA with EUS-FNA(a) or EUS-B-FNA(b).

The summary diagnostic odds ratio (DOR) for the combination of EBUS-TBNA with EUS-FNA was 413.39 (95%CI 179.99 to 949.48) (Figure 1a), higher than the DOR for the combination of EBUS-TBNA with EUS-B-FNA (256.38, 95%CI 45.48 to 1445.32) (Figure 1b). Similarly, the area under the summary receiver operator characteristic (SROC) curve was 0.99 (95%CI 0.98 to 1.00; Figure 2a) when EBUS-TBNA was combined with EUS-FNA, higher than when it was scombined with EUS-B-FNA (0.97, 95%CI 0.92 to 1.00; Figure 2b).

### Publication bias

**Figure 3:**
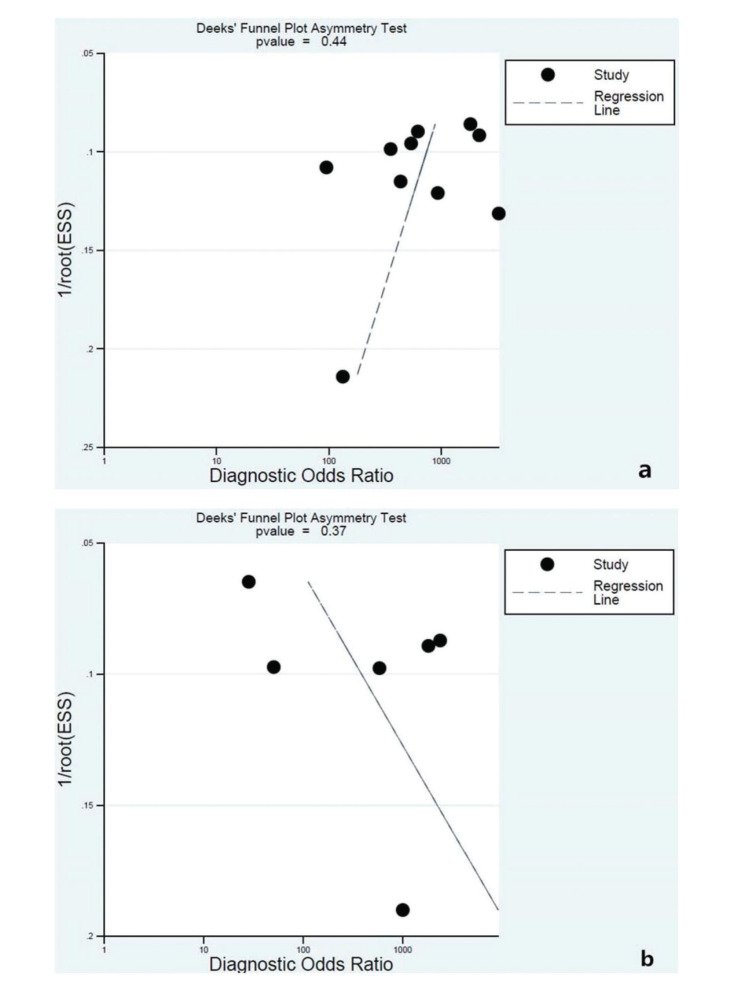
Funnel plot to detect publication bias among studies involving the combination of EBUS-TBNA with EUS-FNA(a) or EUS-B-FNA(b).

Funnel plots of sensitivity as a function of sample size were symmetrical for the two modality combinations (Figures 3), suggesting no significant publication bias.

## DISCUSSION

Mediastinoscopy is considered the gold standard for diagnosis and staging of mediastinal masses and lymph nodes (14). However, it is an invasive technique that requires general anesthesia, cannot evaluate all the mediastinal and hilar lymph nodes (14-15), and cannot easily be repeated for restaging (16). The available literature suggests that combining EBUS-TBNA with either EUS-FNA or EUS-B-FNA provides relatively high accuracy when diagnosing mediastinal diseases, while the combination with EUS-FNA may be slightly better. Our analysis provides the first systematic support for recent guidelines (8-10) recommending the combination of EBUS-TBNA with either EUS-FNA or EUS-B-FNA over either test alone to diagnose and stage mediastinal diseases in a minimally invasive way.

Generally, EBUS-TBNA is used for real-time imaging and aspiration biopsy of mediastinal and hilar masses (in stations 2-4, 7, 10, and 11), while EUS-FNA is used to assess the posteroinferior mediastinum (in stations 4L, 5, and 7-9). Since the first report (17) of the combination of EBUS-TBNA and EUS-FNA for mediastinal staging, several studies (18-26) have found that it can provide high sensitivity and specificity, which we confirm in this pooled analysis. As another advantage, this modality combination is more cost-effective than either EBUS-TBNA or EUS-FNA alone (27).

On the other hand, this modality combination requires using both a bronchoscope for EBUS and an endoscope for EUS. A simpler, faster alternative is to combine EBUS-TBNA with EUS-B-FNA (6), which means that one clinician can perform all procedures using an EBUS bronchoscope. Our meta-analysis of published researches (28-33) indicates that this modality combination also allows high diagnostic accuracy, although potentially less than with EUS-FNA. As we showed heterogeneity in six studies of combined EBUS-TBNA with EUS-B-FNA, it is not rare in systematic reviews of diagnostic accuracy studies. The causes mainly are variability in the patient and study characteristics (34). In this meta-analysis, compared with the combined EBUS-TBNA and EUS-FNA, the factors that mainly contributed to the high heterogeneity are the technical proficiency and the research quantity. We did not take these issues into account because this technology is not widely used over the world, and the studies are too rare to extract.

The combined methods of EBUS-TBNA with EUS-FNA or EUS-B-FNA are suitable for diagnosis and staging of mediastinal diseases, and EUS-B-FNA is more appropriate for patients with poor lung function. The usefulness of combining modalities for diagnosis and staging of a mediastinal disease poses a practical challenge since the clinician performing the techniques requires the skills and experience of both a pulmonologist and gastroenterologist. Experienced pulmonologists can safely and accurately perform EUS-B-FNA, and thereby detect lesions inside and outside the lymph nodes with high sensitivity (35), but pulmonologists are not routinely trained to perform EUS. In addition, EUS and EBUS instruments are not typically located together in hospitals. It is probably no coincidence that four of the six studies of combined EBUS-TBNA and EUS-B-FNA in our meta-analysis were published only within the last five years (30-33). At present, combined EBUS-TBNA and EUS-FNA maybe have better diagnostic efficiency for mediastinal diseases, but considering the advantages of combined EBUS-TBNA and EUS-B-FNA, medical schools and healthcare institutions may need to revise training programs for pulmonologists in light of official guidelines, which the present meta-analysis validates.

## AUTHOR CONTRIBUTIONS

This research was initiated by Shen Y, and was completed by Shen Y and Qin S. The manuscript was written by Shen Y and Jiang H revised it.

## Figures and Tables

**Table 1:** Summary of included studies.

Study/year	Patient Numbers	Method	TP	FP	FN	TN	SEN	SPE	PPV	NPV
Vilmann P et al. (18)	28	EUS-FNA+EBUS-TBNA	28	0	0	28	1.00	1.00	1.00	1.00
Wallace MB et al. (19)	138	EBUS-TBNA+EUS-FNA	39	0	3	96	0.93	1.00	1.00	0.97
Szlubowski A et al. (20)	120	EUS-FNA+EBUS-TBNA	19	2	9	90	0.68	0.98	0.90	0.91
Annema JT et al. (21)	123	EUS-FNA+EBUS-TBNA	56	0	10	57	0.85	1.00	1.00	0.85
Herth FJ et al. (28)	139	EBUS-TBNA+EUS-B-FNA	72	0	3	57	0.96	1.00	1.00	0.95
Hwangbo B et al. (29)	143	EBUS-TBNA+EUS-B-FNA	41	0	4	98	0.91	1.00	1.00	0.96
Ohnishi R et al. (22)	110	EBUS-TBNA+EUS-FNA	28	0	11	71	0.72	1.00	1.00	0.87
Szlubowski A et al. (23)	110	EUS-FNA+EBUS-TBNA	55	1	5	49	0.92	0.98	0.98	0.91
Kang HJ (group A) et al. (24)	74	EBUS-TBNA+EUS-FNA	29	0	5	40	0.85	1.00	1.00	0.89
Kang HJ (group B) et al. (24)	74	EUS-FNA+EBUS-TBNA	23	0	2	49	0.92	1.00	1.00	0.96
Liberman M et al. (25)	166	EBUS-TBNA+EUS-FNA	41	0	5	120	0.89	1.00	1.00	0.96
Lee KJ et al. (30)	37	EBUS-TBNA+EUS-B-FNA	29	0	0	8	1.00	1.00	1.00	1.00
Oki M et al. (31)	146	EBUS-TBNA+EUS-B-FNA	24	0	9	113	0.73	1.00	1.00	0.93
Szlubowski A et al. (32)	106	EBUS-TBNA+EUS-B-FNA	38	2	18	48	0.68	0.96	0.95	0.73
Crombag LMM et al. (33)	244	EBUS-TBNA+EUS-B-FNA	84	19	19	122	0.82	0.87	0.82	0.87
Tutar N et al. (26)	20	EBUS-TBNA+EUS-FNA	10	0	1	9	0.91	1.00	1.00	0.90

FN, number of false negatives; FP, number of false positives; NPV, negative predictive value; PPV, positive predictive value; SEN, sensitivity; SPE, specificity; TN, number of true negatives; TP, number of true positives.
