# Erratum for: Endobronchial ultrasound-guided transbronchial needle aspiration combined with either endoscopic ultrasound-guided fine-needle aspiration or endoscopic ultrasound using the EBUS scope-guided fine-needle aspiration for diagnosing and staging mediastinal diseases: a systematic review and meta-analysis

**DOI:** 10.6061/clinics/2020/e1759err

**Published:** 2020-11-02

CLINICS 2020;75:e1759err

Erratum for: doi: https://doi.org/10.6061/clinics/2020/e1759, published in 2020.

Replace the corresponding author: Yanhua Shen

For: Haixing Jiang*

Replace the corresponding email: shenyanhua99@163.com

For: jihaxi@163.com

